# Prevalence of ideal cardiovascular health in the Brazilian adult population - National Health Survey 2019

**DOI:** 10.1590/S2237-96222023000300006

**Published:** 2023-04-03

**Authors:** Ana Carolina Souto Valente Motta, Kelb Bousquet-Santos, Isabela Harumi Lopes Motoki, Joanlise Marco De Leon Andrade

**Affiliations:** 1 Universidade de Brasília, Departamento de Estatística, Brasília, DF, Brazil; 2 Universidade de Brasília, Colegiado de Bases Biológicas e da Saúde, Brasília, DF, Brazil

**Keywords:** Population Based Studies in Public Health, National Health Survey, Risk Factors of Cardiovascular Disease, Health Inequality, Cross-sectional Studies, Estudios Poblacionales en Salud Pública, Encuesta de Salud, Factores de Riesgo de Enfermedad Cardiovascular, Desigualdad en Salud, Tasa de Prevalencia, Epidemiología Descriptiva, Estudios Transversales, Estudos Populacionais em Saúde Pública, Inquérito Nacional de Saúde, Fatores de Risco de Doenças Cardíacas, Desigualdade em Saúde, Estudos Transversais.

## Abstract

**Objective:**

to analyze the prevalence of ideal cardiovascular health (CVH) in the Brazilian adult population based on the 2019 National Health Survey.

**Methods:**

this was a population-based cross-sectional study (n = 77,494); prevalence and respective 95% confidence intervals (95%CI) of ideal CVH (seven metrics achieved simultaneously) and by individual metrics (four behavioral and three biological metrics), as defined by the American Heart Association, were estimated.

**Results:**

only 0.5% (95%CI 0.4;0.6) of the study population presented ideal CVH, with higher prevalence among those with higher level of education (1.3%; 95%CI 0.9;1.6) and residents in urban areas (0.6%; 95%CI 0.5;0.7); the prevalence of behavioral and biological metrics was 0.7% (95%CI 0.6;0.8) and 63.3% (95%CI 62.7;63.9) respectively.

**Conclusion:**

the prevalence of ideal CVH was very low, highlighting the need for public policies aimed at promotion, surveillance and CVH care in the Brazilian adult population.

## Resumo

**Objetivo:**

analisar a prevalência de saúde cardiovascular (SCV) ideal na população adulta brasileira.

**Métodos:**

estudo transversal com base na Pesquisa Nacional de Saúde de 2019 (n = 77.494); foram estimadas as prevalências e respectivos intervalos de confiança de 95% (IC_95%_) de SCV ideal (sete metas alcançadas simultaneamente) e por metas individuais (quatro metas comportamentais; três biológicas), conforme propõe a Associação Americana do Coração.

**Resultados:**

apenas 0,5% (IC_95%_ 0,4;0,6) da população estudada apresentou SCV ideal, observando-se maior prevalência entre aqueles com maior escolaridade (1,3%; IC_95%_ 0,9;1,6) e os residentes em áreas urbanas (0,6%; IC_95%_ 0,5;0,7); as prevalências das metas comportamentais e biológicas foram de 0,7% (IC_95%_ 0,6;0,8) e 63,3% (IC_95%_ 62,7;63,9) respectivamente.

**Conclusão:**

a prevalência de SCV ideal foi muito baixa, evidenciando a necessidade de políticas públicas para promoção, vigilância e atenção à SCV na população adulta brasileira.

## Resumen

**Objetivo:**

analizar la prevalencia de salud cardiovascular (SCV) ideal en la población adulta brasileña con base en la Encuesta Nacional de Salud de 2019.

**Métodos:**

estudio transversal de base poblacional (n = 77.495). Según lo propuesto por la Asociación Americana del Corazón, la prevalencia y los intervalos de confianza del 95% (IC_95%_) del SCV ideal se estimaron globalmente (siete metas alcanzadas simultáneamente) y por metas individuales (cuatro metas de comportamiento y tres metas). Se calculó la prevalencia de las metas por variables sociodemográficas.

**Resultados:**

sólo el 0,5% (IC_95%_ 0,4;0,6) de la población presentó SCV ideal, siendo más frecuente en aquellos con educación superior (1,3%; IC_95%_ 0,9;1,6) y entre los residentes de áreas urbanas (0,6%; IC_95%_0,5;0,7). La prevalencia de las metas de comportamiento y biológicas fue de 0,7% (IC_95%_ 0,6;0,8) y 63,3% (IC_95%_ 62,7;63,9), respectivamente.

**Conclusión:**

la prevalencia de SCV ideal en adultos brasileños es muy baja, destacando la necesidad de políticas públicas para la promoción, vigilancia y atención a la SCV en la población adulta brasileña.

**Table:** 

Study contributions	
**Main results**	The prevalence of ideal cardiovascular health (CVH) was 0.5% in Brazil in 2019. The prevalence of behavioral and biological CVH metrics was 0.7% and 63.3% respectively. Diet metric showed the lowest prevalence in the study population (4.3%).
**Implications for services**	The results obtained can contribute to the development of public policies aimed at health promotion, surveillance and care. The prevalence estimates of the CVH metrics indicate which targets and population segments efforts should be directed to.
**Perspectives**	Topics for future studies include estimating the prevalence of CVH metrics for population subgroups, identifying demographic and socioeconomic variables that affect that prevalence, and evaluating new CVH criteria.

## INTRODUCTION

Cardiovascular diseases (CVDs) are the leading cause of death globally and in Brazil, accounting for one third of all deaths.^1^ Among CVDs, coronary artery disease was the leading cause of death in the country.^2^ In the period from 1990 to 2019, there was a reduction in the age-adjusted CVD mortality rate in all Federative Units (FUs), although less significant in the North and Northeast, when compared to the other regions.^2^

Modifiable risk factors account for 70% of CVD deaths.^3^ Unhealthy diet, obesity, sedentary lifestyle and tobacco use represent behavioral risk factors, while metabolic factors include high cholesterol levels, arterial hypertension and diabetes *mellitus*. In several countries, the identification of the most prevalent CVDs risk factors in the population has allowed cardiovascular disease prevention programs to significantly reduce mortality from CVD.^4^ In Brazil, different data sources enable continuous monitoring of mortality, morbidity and risk factors for CVD, such as the Mortality Information System (*Sistema de Informações sobre Mortalidade* - SIM), the Hospital Information System of the Brazilian National Health System (*Sistema de Informações Hospitalares do Sistema Único de Saúde* - SIH/SUS) and periodic health surveys, such as the National Health Survey (*Pesquisa Nacional de Saúde* - PNS).^2^

In 2010, the American Heart Association proposed the concept of “ideal cardiovascular health” aimed to improve the cardiovascular health of the American population and reduce deaths from CVD.^5^ Based on the premise of primary prevention, the seven-component metrics of the CVH score are divided into three biological metrics - adequate blood pressure, cholesterol and serum glucose levels - and four behavioral metrics - healthy diet, physical activity, ideal body weight and non-smoking. Since its creation in 2010, the CVH score has been widely used in the international scientific literature, in association with cardiovascular outcomes or other types of outcomes.^6^ ^)-(^ ^8^

The prevalence of ideal CVH in the Brazilian population, taking into consideration the 2013 PNS data, was less than 1%, being lower in men, in individuals aged 60 years and older and among residents of the country Southern region.^9^ Similar results were obtained in international studies,^10^ ^),(^ ^11^ which showed a prevalence of CVH of less than 1% and null, in the Southern Cone countries of South America (Argentina, Chile and Uruguay) and in the United States, respectively.^12^ ^)-(^ ^14^

Given the very low prevalence of ideal CVH in Brazil in 2013, and the fact that the CVH score has been a useful tool for cardiovascular surveillance, it can be concluded that the monitoring of CVH indicators, represented by the biological and behavioral metrics of the CVH score, constitute a necessary strategy for the primary prevention of CVDs. However, until the conclusion of this article, we could not find more recent published studies that have evaluated the prevalence of ideal CVH in a representative sample of the Brazilian population. The objective of this study was to analyze the prevalence of ideal CVH in the Brazilian adult population.

## METHODS

*Study design*

This was a cross-sectional study using 2019 PNS data aimed to determine the prevalence of individuals with ideal CVH in the Brazilian adult population. The prevalence and respective 95% confidence intervals (95%CI) were calculated for the CVH metrics, as well as by variables of sociodemographic characteristics.

*Setting*

The PNS is a nationwide, household-based health survey conducted by the Ministry of Health in partnership with the Brazilian Institute of Geography and Statistics (*Instituto Brasileiro de Geografia e Estatística* - IBGE) in 2013 and 2019.^15^ ^)^ The study population is comprised of residents of permanent private households in Brazil, with the exception of those located in the special census tracts.^15^ The PNS sample is a subsample of the Master Sample of the National Household Sample Survey (*Pesquisa Nacional por Amostra de Domicílios* - PNAD), with advances in both geographical spread and estimate accuracy.^15^

Regarding sample selection, the primary sampling units (PSUs) were selected by simple random sampling, maintaining the stratification of the master sample of the PNAD. Thus, a fixed number of permanent private households were randomly selected in each of these PSUs from the National Register of Addresses for Statistical Purposes (*Cadastro Nacional de Endereços para Fins Estatísticos* - CNEFE), totaling 108,525 households. Finally, a resident aged 15 years and older was selected in each household of the sample, based on the list of eligible household residents obtained at the time of the interview, in order to answer the individual interview. The final sample was comprised of a total of 94,114 individual interviews conducted. After filling out a list of all individuals living in the household, the interviewer identified the resident who would provide the information about the household and all the residents’ questionnaires, in addition to randomly select the resident aged 15 years or older to answer the individual interview. The selected resident’s questionnaire had questions related to lifestyles, chronic non-communicable diseases (NCDs), among others. Further details about the study design can be found in the literature.^15^ ^),(^ ^16^

The calculation of the sample size of households and people took into consideration (i) the various indicators of interest, (ii) proportion estimation with a 95%CI, (iii) the effect of the sampling plan, (iv) the number of households selected by PSU, (v) the proportion of households with people in the age group of interest and (vi) the possibility of estimating the main indicators at the state and state capital level.^15^

*Participants*

The exclusion criteria of the present study were (i) age under 18 years old, (ii) incomplete individual interviews, (iii) individual questionnaires answered by third parties, (iv) pregnant women or women who did not know whether they were pregnant and (v) missing response of one or more variables of CVH. The final sample for this study was comprised of 77,494 individuals.

*Variables*

a) Cardiovascular health (CVH)

In this study, the CVH metrics evaluated were adapted from the original metrics defined by the American Heart Association in 2010.^5^ The four behavioral metrics and the three biological metrics were evaluated based on self-reported information, as shown in Box 1. Each metric (variable) was categorized as favorable outcome (= 1) or unfavorable outcome (= 0). The CVH score was obtained from the sum of the results of the seven metrics, ranging from 0 to 7. Ideal CVH was reached when the individual presented a CVH score = 7, that is, when he/she obtained a favorable outcome (= 1) in the seven metrics (Box 1).^5^ The original codes (indicated in the dictionary of 2019 PNS)^17^ of the variables used and the results considered favorable are available in the supplementary material attached to this manuscript.^5^ Whole grain intake was not considered for the ideal diet metric as this information was not collected by 2019 PNS.

b) Sociodemographic

- sex (male; female);

- region of Brazil (North; Northeast; Midwest; Southeast; South);

- Urban-Rural status (urban; rural);

- age group (in years: 18 to 24; 25 to 39; 40 to 59; 60 or more);

- race/skin color (Mixed race; White; Black; Asian/Indigenous/ignored);

- marital status (single; married; separated/divorced/widowed);

- level of education (up to incomplete elementary school; from complete elementary school to incomplete high school; from complete high school to incomplete higher education; complete higher education); and

- occupation (employed, unemployed).

c) Chronic diseases

A variable “chronic diseases” was defined when an individual reported at least one of the following chronic diseases: asthma (or asthmatic bronchitis); arthritis or rheumatism; chronic back or neck pain, low back pain, sciatica and spinal disc problems; work-related musculoskeletal disorders; cardiovascular diseases; lung disease or chronic obstructive pulmonary disease; cancer; chronic kidney disease; depression; mental illness, such as schizophrenia, bipolar disorder, psychosis or obsessive compulsive disorder; chronic disease in general (physical or mental) or long-term illness (more than 6 months).

*Data sources/measurement*

The data are public and available on IBGE^15^ and PNS website (https://www.pns.icict.fiocruz.br/bases-de-dados/).

*Statistical methods*

Initially, descriptive analyses of the study population characteristics were performed, calculating proportions (for categorical variables), in addition to mean and standard deviation (for continuous variables). Subsequently, the prevalence and respective 95%CI were estimated for the following conditions: (i) ideal CVH; (ii) CVH score (0-7 metrics); (iii) behavioral metrics and their respective score (0-4); (iv) biological metrics and their respective score (0-3). Finally, the prevalence of ideal CVH and behavioral and biological metrics were described according to sociodemographic characteristics and the presence of chronic disease.^15^

Differences in proportions were considered statistically significant when there was no overlap in the respective confidence intervals.^18^ The analyses were performed using the software R^19^, version 3.6.2. The survey package was used for adjustment by complex sampling and the study design of 2019 PNS.^15^

*Ethical aspects*

The PNS 2019 was approved by the National Research Ethics Committee (*Comissão Nacional de Ética em Pesquisa* - CONEP) of the National Health Council (*Conselho Nacional de Saúde* - CNS) in August 2019: Opinion No. 3,529,376.^15^ The research complied with CNS Resolution No. 196 of October 10, 1996, ensuring voluntariness, anonymity and possibility of withdrawal at any time from the study for the research subjects.

**Box 1:** Cardiovascular health metrics adapted from the American Heart Association

Metrics	Metric used
Behavioral	4 behavioral metrics achieved simultaneously
Smoking	Never smoked or quit smoking for more than 12 months.
Body mass index (BMI)	< 25 kg/m² (based on self-reported weight and height).
Physical activity	exercises or sports ≥ 150min./week
Healthy diet	4 diet metrics achieveds simultaneously:
	i) fruits and vegetables: consumption 1 or more times/day;
	ii) fish: consumption ≥ twice/week;
	iii) sodium: salt intake in freshly prepared food and processed foods in adequate, low or very low amounts; and
	iv) sugar: consumption of soft drinks and/or industrialized fruit juices < 5 days/week.
Biological	3 biological metrics achieved simultaneously
Total cholesterol	Absence of self-reported medical diagnosis of high cholesterol.
Blood pressure	Absence of self-reported medical diagnosis of arterial hypertension.
Glucose	Absence of self-reported medical diagnosis of diabetes *mellitus*.

## RESULTS

The final sample of the study was comprised of 77,494 individuals, with a mean age of 48 (± 17) years. 2,431 individuals under 18 years old, 837 with incomplete individual interviews, 1,711 individual questionnaires answered by third parties, 3,131 pregnant women or women who did not know whether they were pregnant and 8,508 individuals with missing data for one or more CVH variables, were excluded.

**Figure 1:**
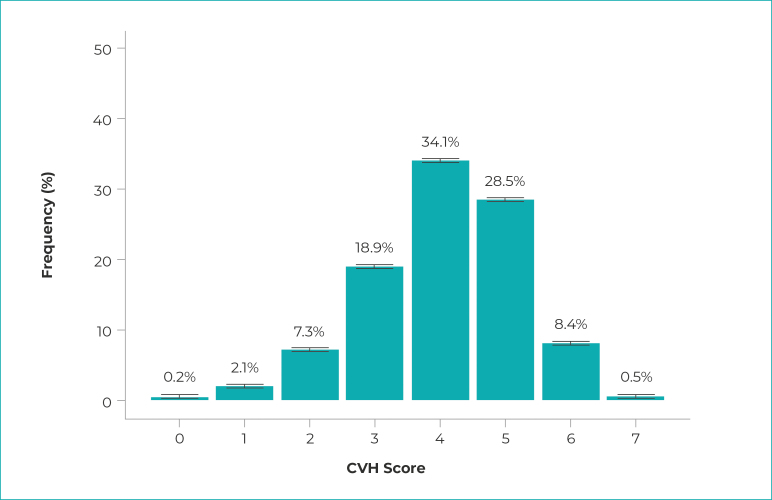
- Distribution of population frequency according to the values obtained in the cardiovascular health (CVH) score^a^ with respective 95% confidence intervals, estimated for the adult population, Brazil, 2019

The sociodemographic characteristics of the sample are shown in Table 1. Higher frequencies were observed for female participants (54.7%), of mixed race/skin color (49.5%), without chronic disease (57.7%), with incomplete elementary education (38.5%), employed (59.6%), living in the Northeast region (33.9%), in urban area (79.0%). Figure 1 shows the distribution of the CVH score in the study population. The prevalence of ideal CVH was 0.5% (95%CI 0.4;0.6) in the Brazilian adult population. It could be seen that 8.9% of the participants presented favorable outcomes in 6 to 7 metrics, 81.5% in 3 to 5 metrics and 9.6% in 0 to 2 metrics.

**Table 1:** Characteristics of the adult Brazilian population (n = 77,494), 2019 National Health Survey

Variables	N	%	95%CI^a^
Sex			
Male	63,359,986	45.2	(44.6;45.9)
Female	76,676,523	54.8	(54.1;55.4)
Age group (years)			
18-24	16,797,883	12.0	(11.5;12.5)
25-39	40,026,391	28.6	(28.0;29.2)
40-59	51,822,239	37.0	(36.4;37.6)
≥60	31,389,996	22.4	(21.8;23.0)
Race/skin color			
Mixed race	59,763,814	42.7	(42.0;43.4)
White	62,440,066	44.6	(43.8;45.4)
Black	15,752,118	11.2	(10.8;11.7)
Asian/Indigenous/ignored	2,080,511	1.5	(1.3;1.7)
Marital status			
Single	55,627,974	39.7	(39.0;40.4)
Married	64,089,556	45.8	(45.1;46.5)
Separated/divorced/widowed	20,318,979	14.5	(14.1;14.9)
Schooling (level of education)			
Up to incomplete elementary education	46,739,391	33.4	(32.7;34.1)
Complete elementary education and incomplete high school	19,367,870	13.8	(13.4;14.3)
Complete high school and incomplete higher education	49,745,301	35.5	(34.9;36.2)
Complete higher education	24,183,947	17.3	(16.6;18.0)
Employment situation			
With a job	86,692,821	61.9	(61.3;62.5)
Without a job	53,343,688	38.1	(37.5;38.7)
Chronic disease			
At least one	63,178,438	45.1	(44.4;45.8)
Without disease	76,858,071	54.9	(54.2;55.6)
Region			
North	10,178,075	7.3	(7.0;7.6)
Northeast	35,417,215	25.3	(24.7;25.9)
Southeast	63,001,662	45.0	(44.1;45.9)
South	20,852,598	14.9	(14.4;15.3)
Midwest	10,586,959	7.5	(7.3;7.9)
Urban-Rural status			
Urban	122,412,326	87.4	(87.0;87.8)
Rural	17,624,183	12.6	(12.2;13.0)

Source: National Health Survey 2019.

95%CI: stands for 95% conficence interval.

a) CVH: cardiovascular health; b) 95% confidence intervals for the CVH score were, respectively: 0.1 to 0.2; 1.9 to 2.3; 7.1 to 7.7; 18.4 to 19.4; 33.5 to 34.7; 27.9 to 29.1; 8.0 to 8.8; and 0.5 to 0.6.

Estimated prevalences of ideal CVH and of behavioral and biological metrics is shown in Table 2. This table also shows the results by sociodemographic variables. Higher prevalences of ideal CVH were observed among individuals with complete higher education (1.3%; 95%CI 0.9;1.6), when compared to the other levels of education, and among residents in urban areas (0.6%; 95%CI 0.5;0.7), when compared to residents in rural areas.

**Table 2:** Prevalences (and 95% Confidence Intervals) of Brazilian adults (n = 77,494) with adequate behavioral and biological metrics and ideal cardiovascular health, National Health Survey, Brazil, 2019

Sociodemographic characteristics	Behavioral metrics					metrics Biological				Ideal cardiovascular health (sum of the 7 metrics)
	Behavioral metrics (sum of the 4 metrics)	Non-smoking	Adequate body weight	Physical activity	Healthy diet	Behavioral metrics (sum of the 3 metrics)	Normal cholesterol levels	Normal blood pressure	Normal blood sugar levels	
Total	0.7 (0.6;0.8)	86.0 (85.6;86.5)	34.1 (33.4;34.7)	27.4 (26.8;28.1)	4.3 (4.0;4.5)	63.3 (62.7;63.9)	84.1 (83.6;84.6)	73.0 (72.4;73.6)	91.4 (91.0;91.7)	0.5 (0.4;0.6)
Sex										
Male	0.5 (0.4;0.6)	82.7 (82.0;83.4)	38.3 (37.4;39.2)	29.3 (28.4;30.2)	3.1 (2.8;3.3)	68.1 (67.3;69.0)	87.5 (86.8;88.1)	77.2 (76.4;77.9)	92.3 (91.8;92.8)	0.4 (0.3;0.5)
Female	0.9 (0.8;1.1)	88.8 (88.3;89.3)	41.7 (40.9;42.6)	25.9 (25.1;26.6)	5.2 (4.9;5.6)	59.2 (58.4;60.1)	81.3 (80.6;81.9)	69.6 (68.8;70.3)	90.6 (90.1;91.1)	0.7 (0.5;0.8)
Age group (years)										
18-24	0.5 (0.2;0.9)	86.6 (85.0;88.2)	61.6 (60.0;63.7)	35.7 (33.4;37.9)	1.6 (1.0;2.1)	92.0 (90.8;93.1)	95.8 (94.9;96.7)	96.4 (95.6;97.2)	99.0 (98.6;99.4)	0.5 (0.2;0.9)
25-39	0.6 (0.5;0.8)	86.7 (85.9;87.5)	39.9 (38.6;41.2)	31.3 (30.2;32.4)	2.6 (2.3;2.9)	83.3 (82.4;84.2)	92.6 (91.9;93.3)	90.1 (89.4;90.8)	97.8 (97.5;98.2)	0.6 (0.4;0.7)
40-59	0.8 (0.7;1.1)	84.6 (83.9;85.4)	34.1 (33.2;35.1)	26.4 (25.3;27.4)	5.0 (4.6;5.3)	58.3 (57.2;59.3)	81.2 (80.4;82.0)	70.4 (69.4;71.4)	91.2 (90.7;91.8)	0.6 (0.4;0.7)
≥60	0.8 (0.7;1.0)	87.3 (86.5;88.0)	39.1 (38.1;40.2)	19.9 (18.9;20.9)	6.7 (6.1;7.2)	30.6 (29.6;31.6)	71.8 (70.7;72.9)	43.0 (41.9;44.2)	79.3 (78.4;80.2)	0.3 (0.2;0.4)
Race/skin color										
Mixed race	0.7 (0.5;0.8)	85.4 (84.7;86.1)	(40.5) (39.7;41.3)	26.1 (25.3;26.9)	3.9 (3.6;4.2)	64.6 (63.7;65.4)	85.1 (84.5;85.6)	73.4 (72.6;74.2)	91.5 (91.1;92.0)	0.5 (0.3;0.6)
White	0.9 (0.7;1.0)	87.0(86.3;87.6)	40.2 (39.2;41.3)	29.0 (27.9;30.0)	4.6 (4.2;5.0)	62.4 (61.4;63.3)	82.8 (82.0;83.6)	73.3 (72.4;74.2)	91.4 (90.9;91.9)	0.7 (0.5;0.8)
Black	0.4 (0.2;0.5)	85.0 (83.6;86.3)	37.8 (36.1;39.6)	26.5 (24.9;28.1)	3.9 (3.3;4.5)	62.4 (60.6;64.2)	85.5 (84.2;86.9)	70.5 (68.9;72.1)	91.0 (90.0;91.9)	0.3 (0.2;0.4)
Asian/Indigenous/ignored	1.3 (0.6;2.0)	84.3 (80.6;87.9)	47.7 (41.8;53.7)	27.0 (22.1;31.8)	5.9 (3.6;8.1)	59.7 (53.9;65.4)	83.1 (78.5;87.7)	70.1 (65.0;75.2)	87.9 (84.2;91.7)	0.8 (0.2;1.3)
Marital status										
Single	0.7 (0.5;0.8)	82.4 (81.5;83.2)	47.2 (46.2;48.2)	29.8 (28.8;30.8)	3.1 (2.8;3.4)	76.7 (75.9;77.5)	89.7 (89.1;90.3)	84.0 (83.3;84.7)	95.4 (95.1;95.8)	0.5 (0.4;0.7)
Married	0.8 (0.7;1.0)	89.8 (89.3;90.4)	35.0 (34.1;35.9)	26.9 (26.0;27.8)	4.7 (4.3;5.1)	57.6 (56.7;58.5)	81.7 (81.0;82.5)	68.9 (68.0;69.8)	89.9 (89.4;90.5)	0.5 (0.4;0.7)
Separated/divorced/widowed	0.9 (0.7;1.1)	84.1 (83.1;85.2)	37.4 (36.0;38.8)	22.5 (21.3;23.7)	6.1 (5.5;6.7)	44.5 (43.1;45.9)	76.0 (74.8;77.3)	55.8 (54.4;57.3)	84.7 (83.8;85.7)	0.4 (0.3;0.6)
Schooling (level of education)										
Up to incomplete elementary education	0.4 (0.3;0.5)	81.7 (80.9;82.5)	39.1 (38.1;40.0)	15.6 (14.8;16.3)	3.7 (3.3;4.0)	48.4 (47.4;49.4)	78.7 (77.8;79.5)	58.9 (57.9;59.9)	85.6 (84.9;86.2)	0.2 (0.1;0.3)
Complete elementary education and incomplete high school	0.5 (0.3;0.7)	82.8 (81.5;84.1)	40.4 (38.7;42.0)	22.9 (21.5;24.4)	3.2 (2.7;3.7)	67.3 (65.7;69.0)	87.4 (86.3;88.5)	75.3 (73.9;76.8)	92.6 (91.7;93.4)	0.3 (0.1;0.5)
Complete high school and incomplete higher education	0.8 (0.6;0.9)	88.7 (88.0;89.4)	40.1 (39.8;41.2)	32.0 (31.0;33.1)	3.8 (3.5;4.2)	72.9 (71.9;73.9)	87.7 (86.9;88.5)	81.8 (81.0;82.7)	94.7 (94.2;95.1)	0.6 (0.4;0.7)
Complete higher education	1.7 (1.4;2.1)	91.6 (90.8;92.4)	40.6 (39.0;41.1)	44.5 (43.0;46.0)	7.2 (6.4;7.9)	68.9 (67.5;70.3)	84.4 (83.4;85.5)	80.2 (79.0;81.3)	94.9 (94.3;95.5)	1.3 (0.9;1.6)
Occupation										
Employed	0.7 (0.6;0.8)	86.1 (85.5;86.6)	39.0 (38.2;39.8)	29.6 (28.8;30.4)	3.8 (3.6;4.1)	71.3 (70.5;72.0)	87.1 (86.5;87.7)	80.5 (79.8; 81.1)	94.6 (94.2; 94.9)	0.6 (0.5;0.7)
Unemployed	0.8 (0.7;1.0)	86.0 (85.3;86.7)	42.0 (41.1;43.1)	23.9 (23.0;24.7)	4.9 (4.6;5.3)	50.3 (49.3;51.3)	79.2 (78.4;79.9)	60.8 (59.9; 61.8)	86.2 (85.5;86.8)	0.5 (0.3;0.6)
Chronic disease										
Without disease	0.9 (0.7;1.0)	86.9 (86.3;87.5)	42.6 (41.6;43.5)	29.2 (28.4;30.1)	4.0 (3.7;4.3)	73.3 (72.6;74.1)	90.2 (89.7;90.8)	81.0 (80.3; 81.6)	93.9 (93.5;94.2)	0.7 (0.5;0.8)
With disease	0.7 (0.5;0.8)	85.0 (84.3;85.7)	37.3 (36.4;38.2)	25.2 (24.4;26.0)	4.6 (4.2;4.9)	51.6 (50.1;52.0)	76.6 (75.8;77.4)	63.3 (62.4; 64.2)	88.3 (87.8;88.9)	0.4 (0.3;0.5)
Region										
North	0.7 (0.5;0.9)	89.5 (88.7;90.3)	42.4 (41.6;43.7)	25.9 (24.7;27.1)	4.9 (4.3;5.5)	69.9 (68.6;71.2)	86.3 (85.4;87.2)	79.4 (78.4;80.5)	93.4 (92.8;94.1)	0.4 (0.3;0.6)
Northeast	0.9 (0.7;1.0)	88.4 (87.8;89.1)	42.7 (41.7;43.7)	27.2 (26.4;28.1)	5.4 (5.0;5.8)	63.9 (63.0;64.9)	83.9 (83.2;84.7)	73.4 (72.6;74.2)	91.9 (91.4;92.4)	0.6 (0.4;0.7)
Southeast	0.8 (0.6;0.9)	84.9 (84.1;85.7)	39.8 (38.6;41.0)	28.1 (26.9;29.3)	4.0 (3.6;4.4)	61.5 (60.4;62.6)	83.3 (82.4;84.2)	71.7 (70.7;72.8)	90.7 (90.1;91.4)	0.6 (0.4;0.7)
South	0.6 (0.5;0.8)	83.9 (82.9;85.0)	36.1 (34.7;37.5)	25.1 (23.8;26.4)	3.2 (2.8;3.7)	63.0 (61.6;64.3)	84.7 (83.8;85.7)	72.2 (70.9;73.4)	91.2 (90.5;92.0)	0.5 (0.3;0.6)
Midwest	0.8 (0.5;1.1)	85.9 (84.9;86.9)	40.0 (38.6;41.4)	29.8 (28.4;31.3)	3.6 (3.1;4.2)	65.8 (64.3;67.4)	85.6 (84.4;86.7)	74.6 (73.3;76.0)	91.7 (90.9;92.6)	0.5 (0.3;0.8)
Urban-Rural status										
Urban	0.8 (0.7;0.9)	86.1 (85.6;86.6)	39.5 (38.7;40.2)	29.1 (28.4;29.8)	4.4 (4.2;4.7)	63.2 (62.6;63.9)	83.9 (83.3;84.4)	73.1 (72.5;73.8)	91.2 (90.9;91.6)	0.6 (0.5;0.7)
Rural	0.4 (0.2;0.5)	85.5 (84.5;86.6)	45.3 (44.1;46.6)	15.8 (14.8;16.9)	3.1 (2.7;3.5)	63.4 (62.2;64.6)	85.7 (84.8;86.5)	71.9 (70.8;73.1)	92.5 (91.9;93.1)	0.2 (0.1;0.3)

Prevalence of 63.3% (95%CI 62.7;63.9) was observed among individuals who reported favorable joint biological outcome (3 metrics), and 0.7% (95%CI 0.6;0.8) favorable joint behavioral outcome (4 metrics) in the study population. Regarding biological metrics, individuals who reported normal blood sugar levels (favorable outcome) presented the highest prevalence (91.4%; 95%CI 91.0;91.7), while those who reported normal blood pressure (favorable outcome) showed the lowest prevalence (73.0%; 95%CI 72.4;73.6).

With regard to behavioral metrics, non-smoking presented the highest prevalence (favorable) (86.0%; 95%CI 85.6;86.5), while healthy diet (favorable) (4.3%; 95%CI 4.0;4.5) presented the lowest prevalence among the study population.

## DISCUSSION

The results of this study showed low prevalence of ideal CVH in the Brazilian adult population. Among the components of the ideal CVH score, it was observed a higher prevalence of compliance with biological metrics (63.3%) in the population, when compared to behavioral metrics (0.7%). The healthy diet metric was mainly responsible for both the low prevalence of behavioral metrics and the low prevalence of ideal CVH condition itself. The analysis of the ideal CVH, according to the sociodemographic profile, showed higher levels of prevalence of ideal CVH in the population with higher level of education and among residents in urban areas.

The main limitation of this study is the use of measures based on self-reported information from PNS study participants. The prevalence of individuals who have achieved favorable biological and behavioral metrics may be underestimated, given that self-reported morbidity data, including medical diagnosis of diabetes *mellitus*, hypercholesterolemia, and hypertension, depend on access to health services for diagnosis. Therefore, people with limited access to these services have fewer opportunities for medical diagnosis. However, self-reported data to assess CVH metrics has been used in other studies^20^ ^)-(^ ^22^ and has a good correlation with biological measures.^23^ Another limitation of this study involves the comparison between subgroups of sociodemographic variables that may not have sufficient number of individuals in some categories, such as race/skin color. As positive points of the study, we highlight (i) the fact that it was performed in a representative sample of the Brazilian adult population and (ii) presented updated data on the prevalence of CVH in Brazil.

The concept of ideal CVH is based on a combination of behavioral and biological metrics, which are associated with evidence of CVD-free survival, healthy longevity, reduced morbidity and health costs.^5^ Although achieving 5 to 7 metrics is an outcome associated with a lower risk of CVD incidence, an intermediate protective effect for CVD can be achieved by meeting 3 to 4 metrics of the CVH score.^24^ In this study, 37.4% of the population met 5 to 7 CVH metrics. However, the prevalence of ideal CVH - when the seven metrics included in the score are achieved simultaneously - was less than 1%. These results are consistent with a previous study, which used data from the 2013 PNS.^9^ Despite some methodological differences in the two editions of the survey and in sample collection, the authors of the aforementioned study reported a prevalence of 0.3% (95%CI 0.2;0.5) for ideal CVH in the PNS 2013. International studies also report a low prevalence of ideal CVH in the adult population, with values ranging from 0% to 0.1%.^6^ ^)-(^ ^8^

The results of this study showed that the prevalence of favorable biological metrics in the study population - taking into consideration the simultaneous presence of three metrics - was higher when compared to behavioral metrics - simultaneous presence of four metrics: 63.3% versus 0.7%, respectively. Normal blood glucose level and non-smoking metrics presented the highest proportions in their respective categories, corroborating the results of the PNS 2013.^9^ With regard to smoking, there has been a downward trend in its prevalence among Brazilian adults, in recent decades, which represents a response to the actions developed by the National Policy on Tobacco Control.^25^

As for the normal glucose level metric, it is worth highlighting that the information obtained based on self-report tends to be less accurate. In fact, another study^26^ based on data from the 2019 PNS reported a 24% increase in the prevalence of diabetes *mellitus*, compared to 2013. Multiple factors contributed to this finding, including an increase in the incidence and diagnosis of diabetes. Nevertheless, obesity and poor eating habits stand out as risk factors associated with a higher prevalence of self-reported diagnosis of diabetes *mellitus* and CVDs in Brazil.

Healthy diet metric was mainly responsible for the low prevalence of ideal CVH in this study. This is due to the fact that only 4.3% of the population presented simultaneous consumption of salt, sugar, fish, fruit and vegetables according to the recommendations in the literature. Other national and international studies,^27^ ^),(^ ^28^ found similar results. For example, in the ELSA-Brasil^28^ study, which evaluated employees aged 35 to 74 years, healthy diet metric showed the worst result (1.3%). Adult individuals who adhere to healthy diet recommendations have lower rates of cardiovascular morbidity and mortality^29^, and thus, the results of this study confirm the previous findings about the importance of interventions to promote healthy eating habits, as part of a strategy for the prevention of CVDs.

It can be concluded that the prevalence of Brazilians with ideal CVH was very low. These results, based on the PNS 2019, follow the trend of the PNS 2013 findings. Behavioral CVH metrics, especially healthy diet, showed the worst results. These findings may be useful for developing actions aimed at CVH health and CVDs prevention. The successful implementation of these actions will possibly lead to a reduction in premature deaths from cardiovascular diseases, which is one of the goals of the 2030 Agenda for Sustainable Development, implemented by the United Nations.^30^ Furthermore, as a result of these actions, a reduction in health costs in Brazil is expected, since cardiovascular diseases are responsible for the highest expenditure on hospitalization in the Brazilian National Health System.^2^

## Supplementary Table 1

### - Construction and methods of calculating variables, National Health Survey 2019, Brazil

**Supplementary Table 1:** - Construction and methods of calculating variables, National Health Survey 2019, Brazil

Variables	Description of the variables of the PNS^a^ 2019 questionnaire	Calculation method
Smoking	P050. Do you currently smoke any tobacco products? Answer options: 1-Yes, daily; 2-Yes, less than daily; 3-I do not currently smoke. P052. In the past, did you smoke any tobacco products? Answer options: 1-Yes, daily; 2-Yes, less than daily; 3-Never smoked. P05901. How long they quit smoking. Options: number of years.	Favorable outcome (1) If the individual is a nonsmoker [P050 =3 and P052 =3] or a former smoker for more than one year [P050=3 and P052 = (1 or 2) and P05901 > 1)]
Body mass index (BMI)	P00104. Weight - Final (in kg) Options: 3 integers and 1 decimal place P00404. Height - Final (in cm) Options: 3 integers	Favorable outcome (1) Weight [P00104] divided by Height squared [P00404] < 0.0025
Physical activity	P035. How many days a week do you usually (did you use to) do physical activities or play sports? Options: 1 to 7 days or 0 - Never or less than once a week. P03701. In general, on the day that you exercise or play sports, how long (in hours) does this activity last? Options: number of hours. P03702. In general, on the day that you (used to) exercise or play sports, how long (in minutes) does this activity last? Options: number of minutes.	Favorable outcome (1) Amount of exercise performed in minutes per day [P03701*60+P03702] multiplied by the number of days per week [P035] ≥ 150
Diet	P015. How many days a week do you usually eat fish? Options: 1 to 7 days or 0 - Never or less than once a week. P02001. How many days a week do you usually drink carton/canned juice or powdered fruit juice? Options: 1 to 7 days or 0 - Never or less than once a week. P02002. How many days a week do you usually drink soda? Options: 1 to 7 days or 0 - Never or less than once a week. P018. How many days a week do you usually eat fruit? Options: 1 to 7 days or 0 - Never or less than once a week. P00901. How many days a week do you usually eat at least one type of vegetable (with the exception of potatoes, manioc, cassava, or yam), such as lettuce, tomatoes, green cabbage, carrots, chayote, eggplant, zucchini? Options: 1 to 7 days or 0 - Never or less than once a week. P02601. Considering freshly prepared food and processed foods, do you think your salt intake is: Options: 1-Very high; 2-High; 3-Adequate; 4-Low; 5-Very low.	Favorable outcome (1) Individuals who achieved all 4diet metrics: (i) Consumption of fruits and vegetables every day [P018 = 7 and P00901 = 7]; (ii) Consumption of fish twice a week [P015 ≥ 2]; (iii) Adequate, low, or very low salt intake [P02601≥3]; and (iv) Consumption of soft drinks and/or industrialized fruit juices less than 5 days a week [P02001+P02002 < 5].
Total cholesterol	Q060. Has any doctor ever diagnosed you with high cholesterol? Options: 1-Yes; 2-No.	Favorable outcome (1) Absence of medical diagnosis of high cholesterol [Q060 = 2].
Blood pressure	Q00201. Has any doctor ever diagnosed you with arterial hypertension (high blood pressure)? Options: 1-Yes; 2-No.	Favorable outcome (1) Absence of medical diagnosis of hypertension (high blood pressure) [Q00201 = 2].
Glucose	Q03001. Has any doctor ever diagnosed you with diabetes? Options: 1-Yes; 2-No.	Favorable outcome (1) Absence of medical diagnosis of diabetes [Q03001 = 2].

a) PNS: *Pesquisa Nacional de Saúde* (National Health Survey).
